# Extended radical resection and chest wall reconstruction for a pulmonary sarcomatoid carcinoma: a case report

**DOI:** 10.1186/s40792-024-01866-1

**Published:** 2024-03-18

**Authors:** Yingzhi Zhao, Shaohua Xie, Haoqian Zheng, Kaixin Zhang, Xin Gao, Wenwu Liu, Wei Dai, Hongfan Yu, Qiuling Shi, Bin Hu, Qiang Li, Tianpeng Xie, Xing Wei

**Affiliations:** 1 Department of Thoracic Surgery, Sichuan Clinical Research Center for Cancer, Sichuan Cancer Hospital & Institute, Sichuan Cancer Center, Affiliated Cancer Hospital of the University of Electronic Science and Technology of China, No. 55, Section 4, South Renmin Rd, Chengdu, 610041 China; 2 Graduate School, Chengdu Medical College, Chengdu, Sichuan China; 3 State Key Laboratory of Ultrasound in Medicine and Engineering, College of Biomedical Engineering, Chongqing Medical University, Chongqing, China; 4 School of Public Health, Chongqing Medical University, Chongqing, China

**Keywords:** Pulmonary sarcomatoid carcinoma, Chest wall invasion, Extended radical resection for lung cancer, Chest wall reconstruction

## Abstract

**Background:**

Pulmonary sarcomatoid carcinoma (PSC) is a rare and highly malignant type of non-small cell lung cancer (NSCLC), for which the treatment of choice is surgery. For peripheral PSC growing outward and invading the chest wall, a complete resection of the affected lung lobes and the invaded chest wall can improve long-term prognosis. However, when the extent of the resected chest wall is large, reconstruction is often required to reduce the risk of postoperative complications. Here, we present a case of PSC invading the chest wall treated with successful extended radical resection for lung cancer and chest wall reconstruction.

**Case presentation:**

A 58-year-old male patient with a nodule in the right upper lobe that had been identified on physical examination 2 years before presentation presented to our hospital with a recent cough, expectoration, and chest pain. Imaging revealed a mass in the right upper lobe that had invaded the chest wall. Preoperative puncture pathology revealed poorly differentiated NSCLC. We performed extended radical resection for lung cancer under open surgery and reconstructed the chest wall using stainless steel wire and polypropylene meshes. The procedure was uneventful, and the patient was discharged 7 days postoperatively. Furthermore, the final pathology revealed PSC.

**Conclusions:**

This case underscores the feasibility of surgical R0 resection in patients with PSC with chest wall invasion and no lymph node metastasis, potentially enhancing long-term outcomes. The novel aspect of this case lies in the individualized chest wall reconstruction for a large defect, using cost-effective materials that offered satisfactory structural support and postoperative recovery, thereby providing a valuable reference for similar future surgical interventions.

## Background

Pulmonary sarcomatoid carcinoma (PSC) is a rare, poorly differentiated tumor with a sarcomatoid cell morphology or sarcomatoid components and is considered a sub-category of non-small cell lung cancer (NSCLC). Its incidence ranges from 0.1 to 0.4% of all lung malignancies [1]. PSC typically manifests in older men with a history of heavy tobacco smoking and usually involves the upper lobes of the lung [2]. This tumor type is notable for its aggressive behavior and potential for malignant transformation of epithelial and mesenchymal cells [2]. It is insensitive to conventional chemoradiotherapy, making surgery an important treatment modality [3]. Even in patients with PSC invading the chest wall, complete resection of the primary tumor and the affected chest wall is reportedly beneficial for long-term survival without evidence of lymph node metastasis [4]. However, chest wall resection destroys the stability and airtightness of the thoracic cavity, and resection of a large area of the bony chest wall may even cause chest wall softening, paradoxical breathing, and scoliosis [5, 6]. Therefore, effective chest wall reconstruction is particularly important to reduce surgical complications and improve the quality of life of patients [7]. To the best of our knowledge, no formal guidelines are currently available for treating sarcomatoid carcinoma of the lungs invading the chest wall, and surgical treatment has rarely been reported. Therefore, we report the case of a patient with a right upper lobe mass invading the chest wall who had preoperative puncture pathology suggestive of NSCLC. We performed a complete resection of the right upper lobe and the involved chest wall and successfully reconstructed the chest wall. The final pathological diagnosis was a PSC.

## Case presentation

**Fig. 1:**
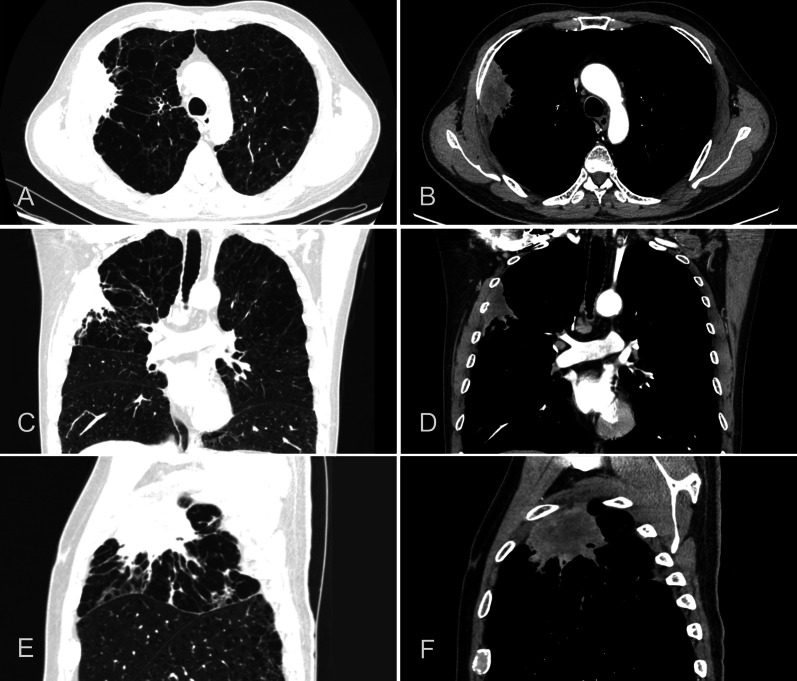
Preoperative enhanced computed tomography scan showing the right upper lobe mass. **A** Cross-section of the lung window; **B** cross-section of the mediastinal window; **C** coronal section of the lung window; **D** coronal section of the mediastinal window; **E** sagittal section of the lung window; **F** sagittal section of the mediastinal window

A 58-year-old male patient with a nodule in the right upper lobe identified on physical examination 2 years before presentation, which was reviewed annually and showed no significant changes, recently presented to our hospital with cough, expectoration, and intermittent pain in the right chest wall. Contrast-enhanced computed tomography (CT) of the chest in our hospital revealed a subpleural soft tissue mass in the apical segment of the right upper lobe, measuring approximately 2.5 × 6.7 cm (Fig. 1), with mild enhancement after contrast agent injection, heterogeneous thickening of the adjacent pleura, and local bone destruction in the anterior segment of the right 4th rib. Fluorine-18-fluorodeoxyglucose positron emission tomography/CT revealed an irregular soft tissue mass shadow under the pleura in the apical segment of the right upper lobe, with an overall range of approximately 67 × 25 mm, and the involvement of the anterior segment of the right 4th rib. Metabolism increased in this lesion with a maximum standardized uptake value of 28.5 and no distant metastasis. The clinical stage was T3N0M0, and the patient underwent a CT-guided lung biopsy the day after admission. Pathological analysis of the biopsy revealed a minimal presence of malignant cells, which were identified as NSCLC (Fig. 4a).

**Fig. 2:**
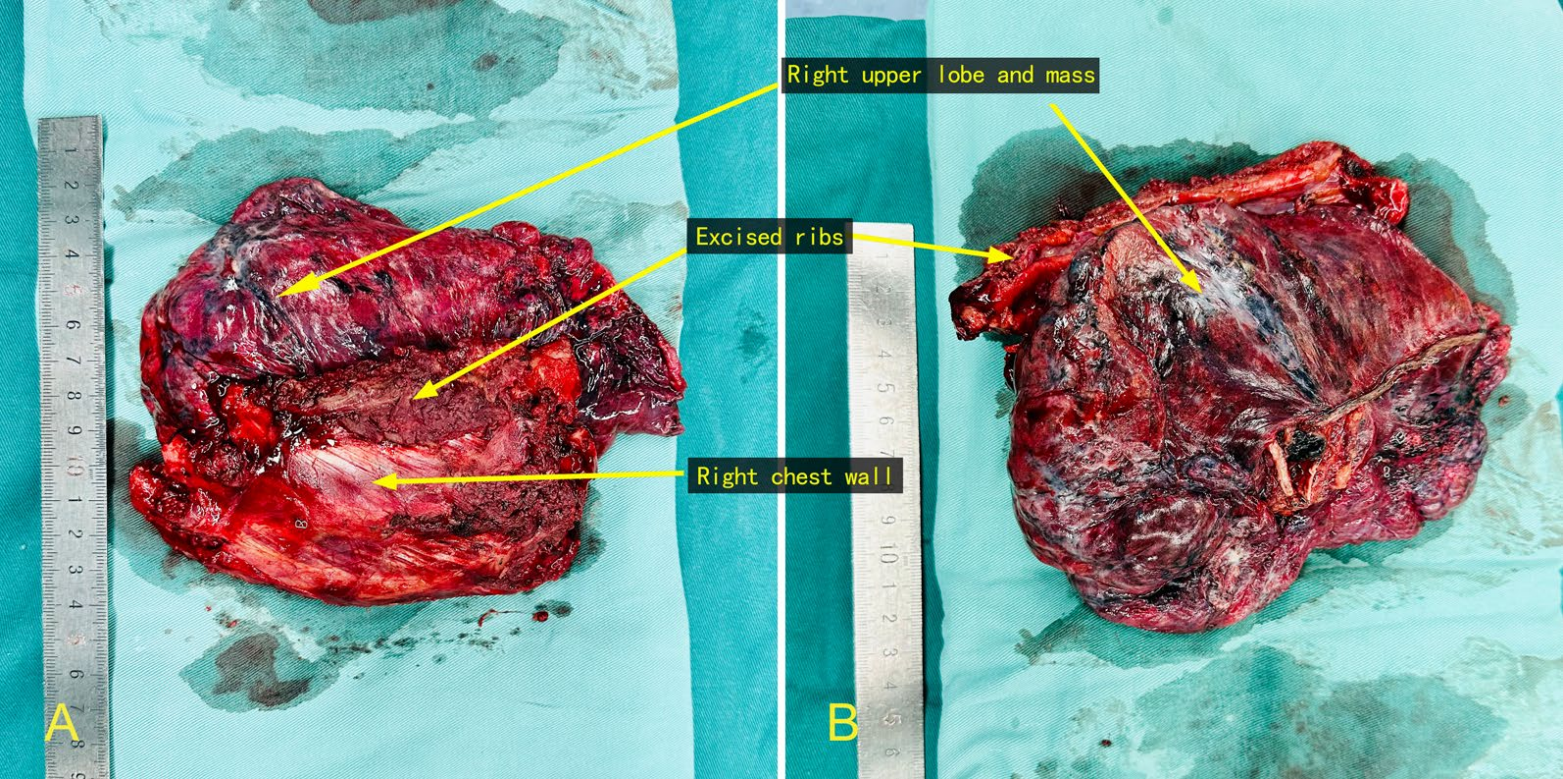
Intraoperative resection of tumor. **A** Chest wall and ribs on the front and lung tissue on the back; **B** lung tissue on the front and chest wall and ribs on the back

**Fig. 3:**
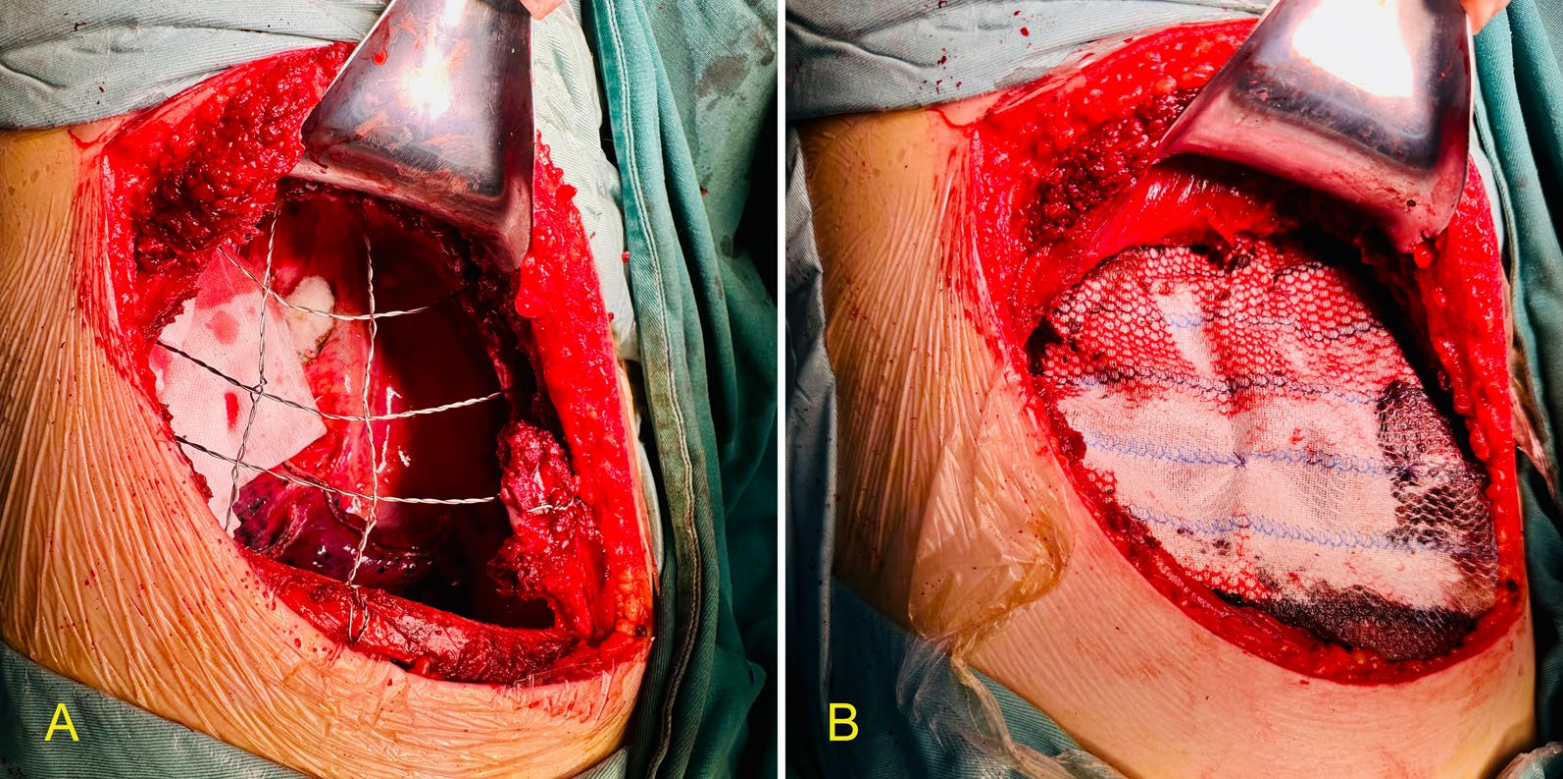
Chest wall reconstruction. **A** Stainless steel wire mesh woven into the chest wall skeleton; **B** polypropylene mesh sutured onto the stainless steel wire mesh

Preoperative examination of the patient showed no significant surgical contraindications. We decided to perform a right upper lobectomy with partial chest wall and rib resection under open surgery to completely resect the lesion without causing seeding dissemination of cancer cells during surgery. Intraoperative chest wall reconstruction was performed according to the size of the resected chest wall. The patient was positioned in the left lateral decubitus position under single-lung ventilation, and the thoracic cavity was opened through an anterolateral incision. Intraoperative findings revealed a mass in the apical segment of the right upper lobe invading the parietal pleura and a suspicious invasion of the right 3rd, 4th, and 5th ribs. An ultrasonic scalpel and an electrocoagulation hook were used to release the adherent pleura. The 3rd, 4th, and 5th ribs and the chest wall were removed to ensure that the incisal margin was at least 2 cm away from the tumor (Fig. 2) [6]. Next, we resected the right upper lobe and systematically dissected the lymph nodes. The chest wall of the patient was resected in the range of approximately 10 × 15 cm; therefore, we decided to perform chest wall reconstruction. Two stainless steel wires were wrapped together and woven into a mesh to improve strength and support. Steel wires parallel to the rib were fixed to the residual end of the rib, and those perpendicular to the rib were fixed to the 2nd and 6th ribs (Fig. 3a). Furthermore, we sutured a polypropylene mesh over the wire mesh to isolate the thoracic cavity (Fig. 3b). A chest drain was placed, and the chest was closed layer-by-layer. The intra-operative bleeding volume and operative time were 100 mL and 210 min, respectively.

**Fig. 4:**
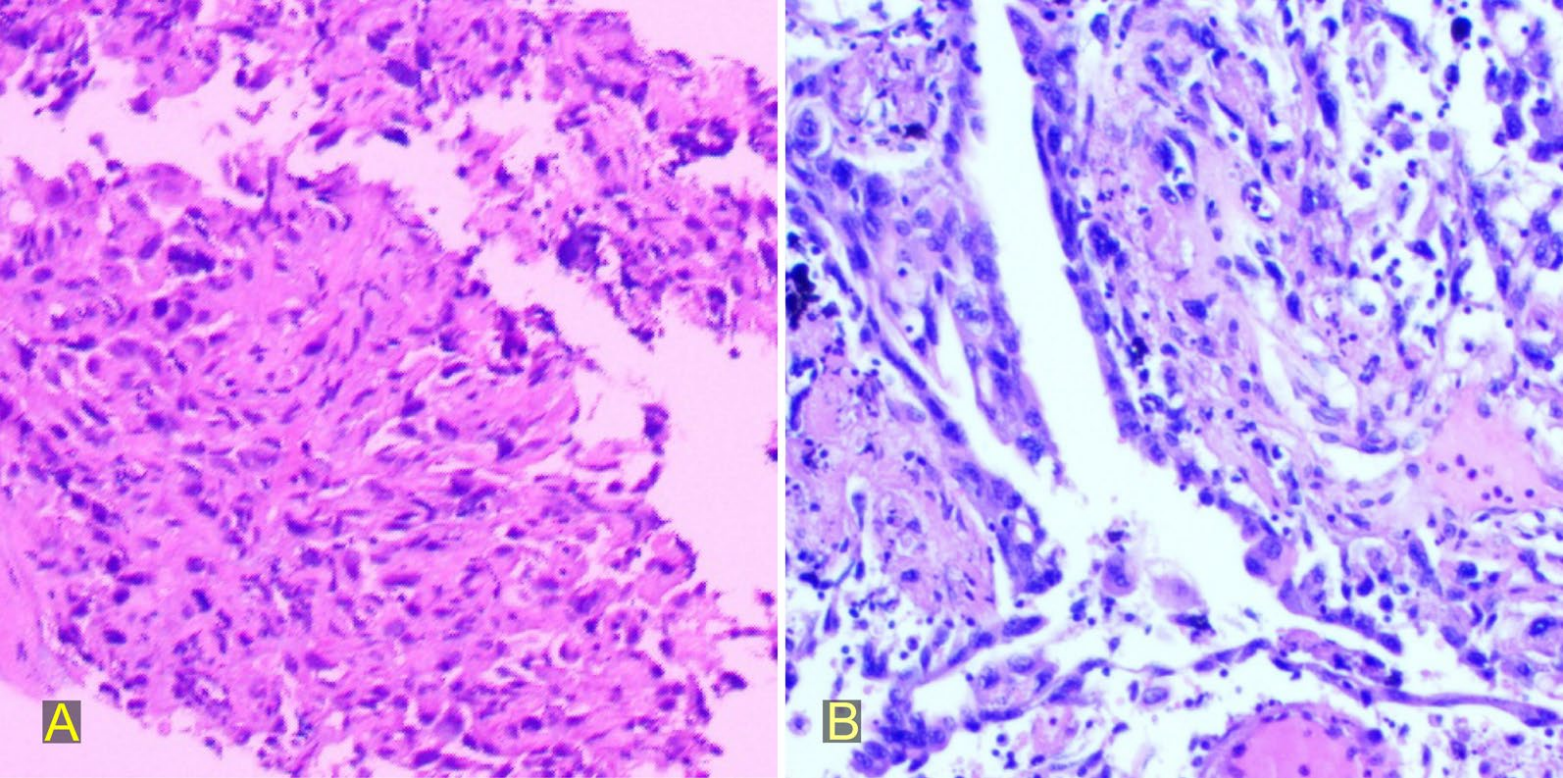
Histological images. **A** Preoperative puncture microscopic images of the tumor (hematoxylin and eosin stain, ×200); **B** postoperative microscopic images of the tumor (hematoxylin and eosin stain, ×200)

At the end of the surgery, the patient was transferred to the intensive care unit and then back to the general ward after his condition stabilized on postoperative day 1. The patient had a pulmonary infection on the afternoon of postoperative day 2. After active anti-infection treatment, the white blood cell count and inflammatory indicators returned to normal on postoperative day 5. The chest tube was also removed on the afternoon of the same day, and the final pathological examination revealed a tumor measuring approximately 8 × 5 cm. It had infiltrated the striated muscles and nerves of the chest wall, although no evidence of rib invasion or lymph node metastasis was observed. The diagnosis of pulmonary sarcomatoid carcinoma was confirmed through a combination of histomorphology and immunohistochemical analysis (Fig. 4b), and the pathological stage was determined to be pT3N0M0. The patient was discharged 1 week postoperatively. At the 2-month follow-up after discharge, the patient had no special discomfort except for occasional pain in the right chest wall. Two cycles of chemotherapy have currently been performed in internal medicine, and the general condition is good.

## Discussion

Sarcomatoid carcinoma of the lungs is insensitive to the current conventional chemoradiotherapy, and surgery is the treatment of choice for PSC [3]. Imaging studies in this patient revealed a right upper lobe mass invading the chest wall, preoperative pathological puncture revealed poorly differentiated NSCLC, and postoperative pathology confirmed PSC. In tumor, node, and metastasis staging, primary lung cancer infiltrating the chest wall is classified as stage T3, regardless of the depth of invasion [6]. Lobectomy and complete resection of the chest wall can be performed for a radical cure in this type of NSCLC, provided no lymph node metastasis exists [8]. Therefore, this patient required extended radical resection for lung cancer even without a preoperative diagnosis of PSC. To better expose the visual field and reduce the risk of surgery, we performed open surgery to remove the lesion while ensuring a tumor-free status. Mazzella et al. [9] reported that incomplete tumor resection, lymph node involvement, and the depth of chest wall invasion are important factors influencing long-term survival for patients with lung cancer and chest wall invasion. Therefore, we performed a right upper lobectomy, systematic lymph node dissection, and extended resection of the chest wall and part of the ribs with a margin distance of > 2 cm to achieve R0 resection in this patient. This treatment choice possibly minimized the likelihood of tumor recurrence and improved long-term survival [4]. The final pathologic findings also supported the rationality of extended resection.

The patient underwent partial excision of the 3rd, 4th, and 5th ribs and the chest wall. The resection site was located between the anterior and midaxillary lines without the support of other bony structures. Studies have shown that anterolateral chest wall defects larger than 5 × 5 cm or rib defects in ≥ 3 areas affect chest wall stability, respiratory function, and circulatory function [10]. Therefore, chest wall reconstruction was necessary for this patient.

Chest wall reconstruction materials may be selected based on surgical approach, facility availability, surgeon preference, and cost-effectiveness. Commonly used rigid chest wall reconstruction materials currently include autologous tissues, biomaterials, and artificial materials. However, autogenous materials were deemed unsuitable for the large chest wall defects in this patient because they are small in size and provide weak support [11]. Biomaterials were also excluded due to their susceptibility to rejection, low tensile strength, and high cost [11]. Artificial materials include titanium alloy products, polymer meshes, and stainless steel wires, among others. Among them, titanium alloy material is the most widely used and was once regarded as the ideal material for chest wall reconstruction. However, recent studies have found that titanium plate has a higher incidence rate of complications after chest wall reconstruction, which can lead to chest wall stiffness, implant breakage, and other risks [12, 13]. Therefore, it should be carefully selected when chest wall reconstruction is performed. Polymer meshes are generally flexible, easily obtainable, and less expensive; however, when solely used for chest wall repair with large defects, insufficient rigidity may occur, which may lead to abnormal respiratory movements [14]. Stainless steel wire has proper compliance and sufficient rigidity, and it provides sufficient support for the chest wall without causing excessive stiffness. It is easily obtainable, less expensive, and easy to popularize. However, to our knowledge, only a few studies have reported its safety and efficacy [15, 16]. Therefore, considering the above factors, we used stainless steel wire and polypropylene meshes as rigid prosthetic support and to cover the wire mesh, respectively. The metal mesh can be fixed around the adjacent ribs or rib stumps; it is resistant to loosening and detachment and will not hinder physiological thoracic movement with appropriate compliance [15]. Furthermore, polypropylene mesh has good water penetration [17]. Subcutaneous and muscular exudates can penetrate the thoracic cavity through metal and polypropylene meshes and flow out from the thoracic drainage tube, preventing the formation of chest wall effusion and hematoma. Currently, placing a drainage tube between each layer of material, which can reduce effusion and drainage tube placement and infections, is not required, thereby reducing the occurrence of postoperative complications. Notably, the implanted wire will move in tandem with the chest wall due to respiration; however, whether prolonged respiratory movement may result in fatigue fracture of the wire is yet to be confirmed through our longer follow-up.

Cases of pulmonary sarcomatoid carcinoma invading the chest wall are uncommon in clinical practice, and reports of extended radical resection in such patients are rare. We report that the patient underwent a successful R0 resection and chest wall reconstruction and was discharged without any complications postoperatively, which may provide an additional reference value for future surgical treatment of sarcomatoid carcinoma of the lungs [18].

## Conclusions

This case suggests that extended radical resection of PSC with chest wall invasion without lymph node metastasis is feasible and can improve the long-term prognosis of patients to some extent. Additionally, based on the economic situation of the patients, we selected less expensive thoracoplasty materials with equivalent effects to alleviate the economic burden on the patient, and this may reduce the occurrence of long-term complications.

## Data Availability

The data and materials are available from the corresponding author upon reasonable request.

## Abbreviations

PSC: Pulmonary sarcomatoid carcinoma
NSCLC: Non-small cell lung cancer
CT: Computed tomography
